# Detection of Anticipatory Dynamics between a Pair of Zebrafish

**DOI:** 10.3390/e26010013

**Published:** 2023-12-21

**Authors:** Wei-Jie Chen, I-Shih Ko, Chi-An Lin, Chun-Jen Chen, Jiun-Shian Wu, C. K. Chan

**Affiliations:** Institute of Physics, Academia Sinica, Taipei 115, Taiwan; alienisme123@gmail.com (W.-J.C.); isis0517@gmail.com (I.-S.K.); linchian73@gmail.com (C.-A.L.); jen.chen@uni-konstanz.de (C.-J.C.); coolneon@gmail.com (J.-S.W.)

**Keywords:** causality, direction of information flow, transfer entropy, anticipatory dynamics, zebrafish

## Abstract

Anticipatory dynamics (AD) is unusual in that responses from an information receiver can appear ahead of triggers from the source, and direction of information flow (DIF) is needed to establish causality. Although it is believed that anticipatory dynamics is important for animals’ survival, natural examples are rare. Time series (trajectories) from a pair of interacting zebrafish are used to look for the existence of AD in natural systems. In order to obtain the DIF between the two trajectories, we have made use of a special experimental design to designate information source. However, we have also used common statistical tools such as Granger causality and transfer entropy to detect DIF. In our experiments, we found that a majority of the fish pairs do not show any anticipatory behaviors and only a few pairs displayed possible AD. Interestingly, for fish in this latter group, they do not display AD all the time. Our findings suggest that the formation of schooling of fish might not need the help of AD, and new tools are needed in the detection of causality in AD system.

## 1. Introduction

Active matters [1] can exhibit different collective phenomena because of their motions and induced interactions with their surroundings. Animals, as a form of active matter, are different from the usual physical active matters in that they can process information to avoid predators and join their mates. One counter-intuitive phenomenon arises from systems capable of information processing is anticipatory dynamics (AD), Ref. [2] in which an information receiving system can produce responses ahead of the information source. It is known that both physical and biological systems are capable of anticipating incoming events. For example, in the phenomenon of anticipating synchronization (AS) proposed by Voss [3], a slave system driven by a master can produce responses ahead of its master in time. Also, our retina [4] can produce responses ahead of predictable stimulation [5]. It is natural to ask if such AD are involved during flocking [6] in birds or schooling [7] in fish, phenomena that are still poorly understood. Presumably, during flocking or schooling, animals do not normally run into each other because they can anticipate the future positions of their group members. Therefore, it is natural to ask if AD can be detected during interactions between moving animals in nature.

If the trajectories of two interacting animals are given as U(t) and V(t) , one would need to know how does one detect the existence of anticipatory dynamics between them. Because anticipatory dynamics is unusual in that causal events between the two time series is not ordered by time, if the events in times series U(t) is found to be ahead of the time series V(t) , there are two possible scenarios: either “ V(t) follows U(t) ” or “ U(t) anticipates V(t) ”. The correct scenario can only be resolved by knowing the direction of information flow (DIF) between them. In this example, if DIF is from U(t) to V(t) , it is the case of anticipation; otherwise, it is just simply the case that later events in V(t) follow earlier events in U(t) . However, the detection of information flow is non-trivial, and tools such as Granger causality, transfer entropy, etc., Ref. [8] have been developed for such purpose.

In this article, we report results of our experiments designed to test for the existence of anticipatory interaction between a pair of interacting zebrafish through their motion trajectories. The method of time lag mutual information (TLMI) [9] is used to first establish the temporal relation between U(t) and V(t) . Then, usual methods of causality are applied to determined the DIF. We found that these methods just confirm the idea that information flows from leading events to following events and therefore do not indicate any AD in experiments. However, by allowing the fish to have a choice between two channels, we were able to determine which fish is in control of the interaction and therefore use it as a indicator of DIF. With this experimental method, we found that a majority of the fish pairs do not show any anticipatory behaviors, and only a few pairs displayed possible AD. Interestingly, for the latter group, the interaction between the fish pair is not always anticipatory. Our result suggests that (a) new tools are needed to detect AD between two time series, and (b) AD might not be needed for the formation of schooling.

## 2. Materials and Methods

In this section, we describe the experimental method and the information theoretic tools to determine the temporal order of the time series and the direction of information flow between them. These methods will help to distinguish between the cases of “anticipate” or “follow” when the two time series are correlated with some time lag. However, this method of anticipatory dynamic detection will fail when there is no significant time lag between the two time series. We will address this problem in Section 4 below.

### 2.1. Experiments of Fish Pairs

In the experiments, a pair of zebrafish is placed in a two-channel rectangular glass tank with the two channels separated by a wall with doors on it. The inner surface of the channels (in contact with water), including the wall, are all opaque and non-reflective, so the fish are not seeing their reflections from these surfaces. The doors allow the fish to choose in which channel to stay and therefore provide a means to detect the DIF between the two fish. The source must be the leading fish who is making the choice, whereas the follower should be on the information receiving end. The rectangular channels are of size 21.6 cm × 14 cm. The fish are 1∼1.5 years old and of length 3.5∼4 cm. The water levels in the two channels are kept around 2 cm deep. This depth was determined empirically to be deep enough to avoid the jumping of fish out of the water and shallow enough to allow the treatment of the channels as one-dimensional.

The tank is placed on top of a LCD monitor (40 cm × 70 cm), which is controlled by a computer to provide different spatially uniform illuminations for the two channels. A CCD camera (Basler, Ahrensburg, Germany acA4096-40um) placed 90 cm above the tank is used to capture images of the fish. An infra-red LED (940 nm) linear array is also used to provide illumination that is visible only to the CCD but not to the fish so that captured image quality can still be maintained even when visible illumination levels in the channels are low. The whole setup is kept inside a box with black walls, preventing the fish from detecting visual cues from the environment.

A total of more than 20 fish were kept in a large tank, and selected fish pairs were then placed into the tank for experiments. We found that a settlement time of 0, 2.5, 5, and 10 min before the start of an experiment recording did not affect the results of our experiments. Trajectories of the two fish during experiments were obtained from video images recorded at 30 or 60 frames per second (fps) with a spatial resolution of 1340×560 square pixels covering an area of 21.6 cm × 9 cm. The recording duration is always set to 500 s. For the health of the fish, the total experiment period per day is limited to 60 min. Trajectories of the fish are extracted from recorded images by the python package: idTracker [10].

**Figure 1:**
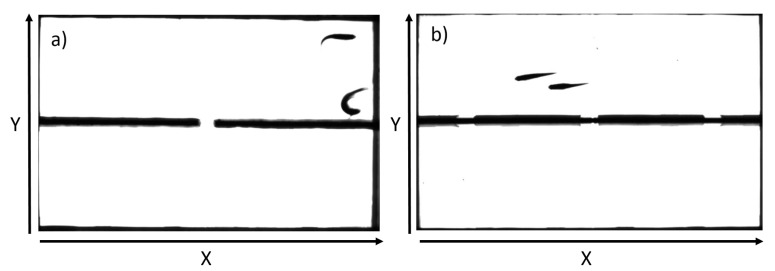
The tank used in the experiment with (**a**) one single door and (**b**) three doors. Directions along the length and the width of the channels are referred to as the X-direction and Y-direction, respectively, as shown in the figure. Note that the walls of the tanks are black.

All experiments were performed at 25 °C. Data reported below were obtained from experiments recorded at 30 fps; data from 60 fps recording gave similar results. Figure 1 shows typical images captured in our experiments. The x-component of the trajectories of one fish will be denoted by $U=\left\{u_{0},u_{1},u_{2}...u_{n}\right\}\equiv \left\{u_{i}\right\}$ with $u_{i}$ being the position of the fish at the *i*th step. The time step size is determined by the frame rate. All the experiments reported below are of step size of 1/30 s. Similarly, $V=\{v_{i}\}$ is defined for the other fish. For the computation of time lag mutual information and transfer entropy described below, $u_{i}$ and $v_{i}$ are discretized to *n* bit resolution so that the horizontal span (x-component) of the tank is divided into $2^{n}$ states. Similarly, the y-component of the trajectories are discretized into two states (1 bit) to indicate in which channel (upper or lower) the fish is located.

### 2.2. Time Lag Mutual Information (TLMI)

As mentioned above, to establish anticipatory dynamics between two time series, we need: (a) the temporal order of events between them and (b) the direction of information flow. Usually, temporal order is the same as as DIF for normal behavior. However, for AD, they should be reversed. To investigate the temporal order between two time series *U* and *V*, we make use of the TLMI, which is defined as:

(1) $$I\left(U,V,\delta t\right)=\sum_{u_{t+\delta t},v_{t}}P\left(u_{t+\delta t},v_{t}\right)log\left[\frac{P(u_{t+\delta t},v_{t})}{P\left(u_{t+\delta t}\right)P\left(v_{t}\right)}\right]$$

where P(…) is the probability distribution or joint distribution of the variables in (…) . It measures how much information is being shared between $\left\{u_{i}\right\}$ and $\left\{v_{i}\right\}$ when *U* is shifted *j* step ( δt=jΔt ) ahead of *V*. Maximum information is being shared between the two time series at the peak of I(U,V,δt) .

The position of the peak in I(U,V,δt) as a function of δt will indicate the relation between similar events between the two time series. If the peak is at positive lag ( $\delta_{p}$ ), it indicates that the time series *V* is leading *U* by an amount of $\delta t_{p}$ . We will label the TLMI between two different time series as the cross-TLMI and that between the same time series as auto-TLMI. Obviously, the peak of the auto-TLMI will always be at zero lag. By comparing the peak heights of auto-TLMI and and cross-TLMI, we will be able to quantify the strength of interaction between the two fish. If there is no sharing of information between the two fish, there will be no peak, and, therefore, peak height equals zero.

### 2.3. Detection of Direction of Information Flow (DIF)

Although the method of time lag mutual information described above can provide the temporal orders of events in the two time series *U* and *V*, it cannot distinguish between “anticipatory” and “following” dynamics. Both the cases of *U* anticipates *V* and *V* follows *U* give the same characteristic peak position in the cross-TLMI between *U* and *V* because in both cases *U* will be leading *V*. The two cases can be separated only when the direction of information flow (DIF) is known. For example, for the case of either *U* anticipates *V* or *V* follows *U*, it is the anticipatory case when information flow from *V* to *U*. Here, we employ three different methods for the detection of information flow. They are described briefly below.

#### 2.3.1. Granger Causality (GC)

Intuitively, if a signal is predictable, one can make guesses about the future of the signal based on its past history. In 1948, Wiener [11] used this idea to predict $u_{i}$ (auto-regression) with constant coefficients $\left\{a_{i}\right\}$ based on $\left\{u_{i-1},u_{i-2},\cdots \right\}$ as

(2) $$u_{i}=\sum_{j=1}^{p}a_{j}u_{i-j}+\epsilon_{i}$$

where *p* is the history length or order of the auto-regression with $\left\{\epsilon_{i}\right\}$ being the error (residuals) of the linear regression model. If *V* is correlated with *U*, one can also use the past of *V* to help for the prediction of the future of *U* with constant coefficients $\left\{b_{i}\right\}$ based on $\left\{v_{i-1},v_{i-2},...\right\}$ as:

(3) $$u_{i}=\sum_{j=1}^{p}a_{j}u_{i-j}+\sum_{j=1}^{p}b_{j}v_{i-j}+\eta_{i}$$

where $\eta_{i}$ are the residuals. The idea of GC is that if the inclusion of *V* can help to improve the prediction of the future of *U*, *U* is said to be GC-caused by *V*. One can then make use of the F-test to establish if $\sum \epsilon_{i}^{2}$ is significantly larger than $\sum \eta_{i}^{2}$ . The F-test will return a *p*-value of the likelihood that the observed difference is caused by chance. Usually, a critical *p*-value of 0.05 is used, and we will use the same value here. Symmetrically, one can also interchange the roles of *U* and *V* to see if *V* is GC-caused by *U*. Here, we used the matlab package MVGC (Multi-Variate Granger Causality) [12] to detect the GC causal relation between *U* and *V*. Note that a quantity related to the F-test is used in MVGC to establish causality; namely $F_{U\to V}$ , which is defined as:

(4) $$F_{V\to U}=log_{e}\frac{|\sum_{\chi \chi }^{\prime }|}{|\sum_{\chi \chi }|}$$

where $\sum_{\chi \chi }^{\prime }=cov\left(\epsilon_{i}\right)$ and $\sum_{\chi \chi }=cov\left(\eta_{i}\right)$ are the residuals covariance matrices of models (2) and (3), respectively. With this method, GC is established as “*U* causes *V*” or DIF from *U* to *V* when $F_{U\to V}>F_{V\to U}$ , and the likelihood is characterized by the corresponding *p*-value.

#### 2.3.2. Liang’s T Method

By considering the rate of information flow between *U* and *V*, Liang [13] derived a tight formula to describe the information flow between *U* and *V* using only the covariance between the four time series *U*, $\dot{U}$ , *V*, and $\dot{V}$ as

(5) $$T_{V\to U}=\frac{C_{UU}C_{UV}C_{V,\dot{U}}-C_{UV}^{2}C_{U,\dot{U}}}{C_{UU}^{2}C_{VV}-C_{UU}C_{UV}^{2}}$$

where $C_{ij}\left(i,j=U,V,\dot{U},\dot{V}\right)$ is the covariance between two time series, with $\dot{U}$ and $\dot{V}$ being the derivatives of *U* and *V*, respectively. Note that $\dot{U}=\{{\dot{u}}_{i}$ }, with ${\dot{u}}_{i}=\frac{u_{i+1}-u_{i}}{\Delta t}$ , and similarly for $\dot{V}$ . With this formulation, Liang was able to show that $T_{V\to U}$ is the maximum likelihood estimator for a linear system. For such a system, if $|T_{V\to U}|$ is non-zero, *V* is causal to *U*; otherwise, it is non-causal.

#### 2.3.3. Transfer Entropy (TE)

We follow the definition of TE of Schreiber [14] and calculate the TE from time series *U* to *V* as

(6) $$T_{U\to V}\left(k,l,u\right)=H\left(V\left(t\right)|V_{t_{-1}}^{\left(k\right)};U_{t_{-1}}^{\left(k\right)}\right)-H\left(V\left(t\right)|V_{t_{-1}}^{\left(k\right)}\right)$$

where H(|) denotes the conditional entropy. The notation $V_{t_{-1}}^{\left(k\right)}=\left\{v_{t-k},v_{t-k+1},...,v_{t-1}\right\}$ represents the history of *V* with a length of *k* time step before the time bin *t* and similarly for *U*. This TE measures the reduction of entropy of the V(t) by using both *V*’s own history and that of *U* with history length *k* and *l* time step, respectively. If $T_{U\to V}$ is positive, we will have information transfer from *U* to *V*. Since there might be information flow in both directions, the net information flow between *U* and *V* is the difference as $\Delta T_{U\to V}\equiv T_{U\to V}-T_{V\to U}$ . If $\Delta T_{U\to V}$ is positive, there will be a net flow of information from *U* to *V*, and vice versa. To simplify analysis, we have set the history lengths in both *U* and *V* to be the same and labeled it as *h*. In the results reported below, we are using the python package PyInform [15].

### 2.4. Simulation of Anticipatory Data

It is known that one can generate anticipatory responses ( Z(t) ) from a predictable signal ( S(t) ) by using a negative group delay (NGD) filter [2]. This NGD model was later improved to understand the response from anticipatory dynamics of a retina [5], and the original model has the following form:

(7) $$\dot{Z}\left(t\right)=-\alpha Z\left(t\right)+k\left(S\left(t\right)-Z\left(t-t_{d}\right)\right)$$

where α is a damping constant, with *k* being the gain of the system. One can think of Z(t) is the response to drive S(t) with a negative feedback ( $Z(t-t_{d})$ ) of delay $t_{d}$ . We will use this model to generate anticipatory responses to verify validity of the causality tests mentioned above. The time series S(t) is sometimes labeled as the master or “driver” signal and as slave or “target” signal for Z(t) .

## 3. Results

### 3.1. Trajectories in One- and Three-Door Tanks

**Figure 2:**
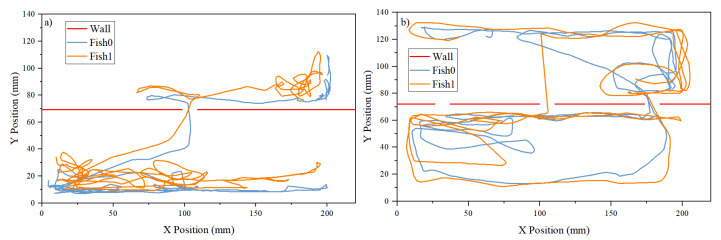
Trajectories of the two fish in (**a**) the tank with one door and (**b**) the tank with three doors. Note that the separation between the two channels is opaque so that the two fish can see each other only through the doors when they are located in different channels. The red horizontal line with openings in the figures indicates the separation between the two channels and the opening of the doors located at *y* = 70 mm. The fish can freely move between the two channels through the doors.

Figure 2a,b show typical trajectories of the two fish in the tank with one and three doors, respectively and Table 1 is the measured probabilities of the choice of doors by the fish. It can be seen that, although there are two channels in the tank, the fish prefer to stay together. That is, when one of the fish choose to move to the other channel, the other fish will follow, indicating that there is some kind of attractive interaction between them. From the figure, it is clear that the main difference between the one-door and three-door tank is that the fish prefer not to use the doors located at the middle of the channel. This last observation can be understood by the observation that the fish spend most of their time at the ends of the channel, as the ends are bounded by three walls. If the probability of going through a door is a random process, the probability of choosing which door to use is then just proportional to the probability of the fish located in the vicinity of the door. Below, we will focus only on the results from the tank with a single door.

**Figure 3:**
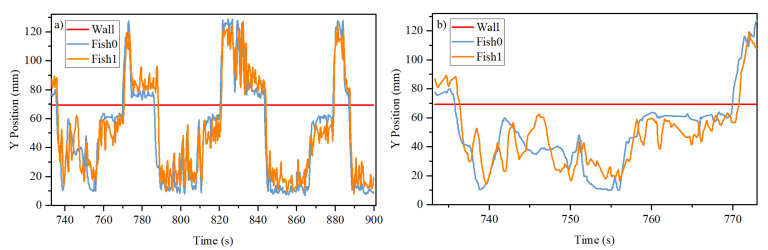
Typical time course of the y-component of the trajectories of the two fish in (**a**) long time scale and (**b**) in short time scale. It can be seen that Fish 1 has faster dynamics, swimming around Fish 0. It also follows Fish 0 from the lower channel to the upper channel. The red horizontal line in the figures indicates the opaque separation between the two channels located at *y* = 70 mm.

**Figure 4:**
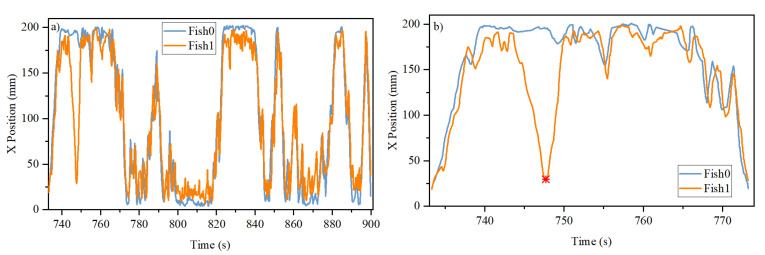
Time course of the x-component of the trajectories shown in Figure 3 of the two fish in (**a**) long time scale and (**b**) short time scale. The “*” marks the time point at which the two fish can be quite far apart.

Since the channels are shaped as a quasi-1D system, we would assume that the dynamics in the X-direction and Y-direction are different. In the X-direction, the fish are interacting naturally. In the Y-direction, it is meaningful in the sense that the fish choose in which channel to stay. Therefore, we decompose the trajectories into their Y-direction time course and X-direction time course as shown in Figure 3 and Figure 4, respectively.

It can be seen from Figure 3 that the two fish are in the same channel most of the time. They are in different channels only briefly, because once one fish starts to move into another channel, the other fish will invariably follow after some short period of time. This phenomenon can be seen as two closely spaced vertical lines in the figure when the fish change their channel. That is, the two fish always stay close together. Sometimes, the two fish can be quite far apart, as shown with the point * in Figure 4b. But very quickly, one of the fish will go the location of the other fish.

### 3.2. Time Lag Mutual Information from the X Component (xTLMI)

**Figure 5:**
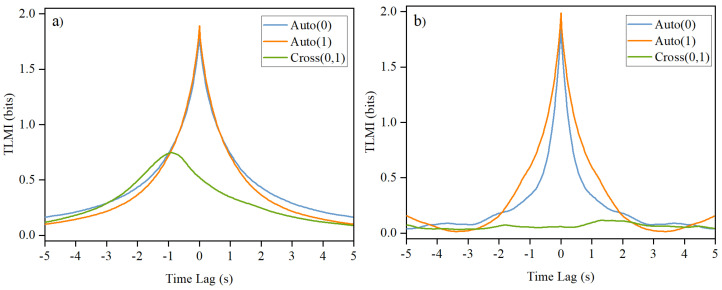
A typical TLMI based on the time course of x-component of the trajectories of the two fish when (**a**) the door in the tank is open and they are almost always in the same channel and (**b**) when the door is blocked and they are in different channels. It can be seen that there is little mutual information when the two fish are in separate channels with a blocked door because they cannot see each other. Auto(0): auto-TLMI of fish 0 and Cross(0,1): cross-TLMI between fish 0 and fish 1. The labels 0 and 1 are arbitrarily assigned to the two fish here.

Figure 5a shows a typical result of the measured xTLMI between the two fish for trajectories similar those shown in Figure 4 when the two fish can swim freely in the tank with the door open. Note that the auto-TLMI always has a peak at zero time lag, and its decay time indicates how fast memory is fading in time. In Figure 5a, the measured auto-TLMI for both fish are similar, indicating that they have similar dynamics. A decay time of the order of second for the auto-TLMI suggests that the trajectories of the fish within a second is strongly correlated. It can also be seen from the figure that the two fish are interacting quite well because the cross-TLMI has a peak height close to 50% of that of the auto-TLMI. The observation that one fish is leading the other fish in the experiments can also be seen from the location of the peak at non-zero time lag.

In order calibrate the effect of interaction strength between the two fish, we have also performed experiments with the door blocked when the two fish were kept in different channels. Figure 5b shows the result of such an experiment. There are two two remarkable differences shown in Figure 5a,b. First, the auto-TLMI for two fish are quite different when they cannot see each other. This observation shows that the interaction between the two fish enable them to have similar dynamics. Second, the cross-TLMI has a prominent peak for the interacting case, whereas it is almost flat and close to zero when there is no interaction. The height of the peak indicates how strongly the two fish interact. Furthermore, we can use Figure 5b as the baseline of mutual interaction between the two fish.

Note that a 2-bit resolution for the computation of TLMI was used in Figure 5 and the theoretical value of the peak height is 2 bit. We have also checked that similar results can be obtained when a 4-bit resolution was used. However, for a 4-bit resolution, the measured results were more noisy because of the increased number of states in the trajectories. Results of xTLMI reported below are all based on 2-bit resolution.

### 3.3. yTLMI and DIF Detection

The results from xTLMI tell us about which fish is leading during their interaction. However, it does not tell us the direction of information flow. Here, we will use properties of the y-component of their trajectories to infer the DIF when they are interacting when the door is open. Since there are two channels in the system and the fish are free to move between them, we will treat the fish as being in two different states (“0” or “1”) when they are in the upper or lower channel, respectively. With this description, the fish changes its state when they go from one channel to the other channel through the door. Our rationale is that the information emitting fish will decide which channel it will take, whereas the information receiving fish will just follow.

**Figure 6:**
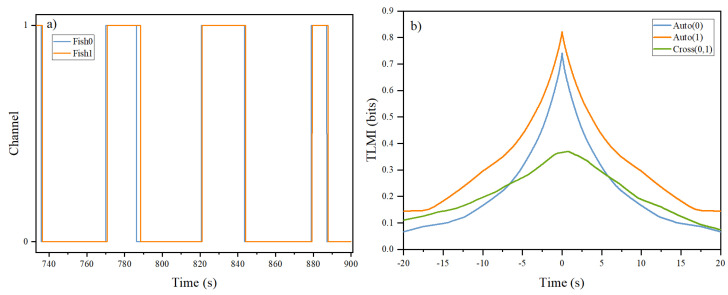
(**a**) A typical time course of the state of the two fish and (**b**) the computed yTLMI based on the time course of the states. The peak position of the yTLMI based on states confirms our observation in (**a**) that Fish 0 changes its state first.

Figure 6a shows the time course of the y-component of the trajectories of the two fish in term of the states of the fish during the experiment. It can be seen that once a fish changed its state, the other fish follows suit. In other words, one of the fish will first decide to which channel it will go and then the other fish will follow. That means the leading fish is the information source and the following fish is the information receiver. If we assume that the role of the fish is constant in time (during experimental recording time), we can then use the yTLMI to determine the direction of information flow with the leader in the yTLMI as the information source. Figure 6b shows a typical result of the computed yTLMI from the time course of the state of the two fish.

One of the main differences between properties of xTLMI and yTLMI is that the decay time scales of their auto-TLMI are very different, as demonstrated by Figure 5 and Figure 6. For xTLMI, the dynamics is dominated by the quasi-1d interaction between the two fish in a single channel. For the yTLMI, the dynamics is dominated by the decision of which channels to take by the leader fish. Intuitively, these two dynamics should be very different. For the xTLMI, the dynamics is determined by how fish interact in short term time scales when they want to be close to each other. As shown in Figure 5, it is on the order of 500 ms. For the yTLMI, the leading fish can choose which channel to take only when it is next to the door. Therefore, the time scale is much longer, on the order of a few seconds, in Figure 6. However, these differences in time scales of auto-TLMI does not impose any limitation to the peak positions of their corresponding cross-TLMI because the peak position of cross-TLMI is determined only by how fast one fish is responding to the other fish.

### 3.4. Summary of Experimental Results

Table 2 is a summary of the important properties of xTLMI and yTLMI obtained from experiments similar to those shown in Figure 5 and Figure 6 above. Here, the peak values of xTLMI (yTLMI) are normalized by the peak height of the corresponding auto-TLMI. With this method, the peak value will be independent of the number of states used in the discretization of the trajectories. As mentioned above, the peak height indicates the strength of interaction between the two fish; we have only included data that have a x-TLMI peak value at least 10% of the peak of the auto-TLMI in the x-component.

When peak position takes a value other than zero, it indicates that one fish is leading the other. Since the labels of the fish are arbitrary, we have chosen the convention that the peak position of the cross y-TLMI is always negative. Therefore, when one also detects a negative peak position in the cross xTLMI, we have the same leading fish in both the x and y components of the trajectories. That means one fish just follows another fish (the case of “following” dynamics). However, when one detects a positive peak position in the cross xTLMI, one would have the situation that the follower in the y-component becomes the leader in the x-component. This is the situation of anticipatory dynamics (AD), because the leading fish in xTLMI is the information receiver while the other fish is the information source. Therefore, with this convention, any fish pair with a positive cross xTLMI peak position is an indication of anticipatory dynamics. For example, in experiment number 5, fish pair D produced a cross yTLMI with peak position at −3.3 s whereas the peak position of cross xTLMI is located at +0.5 s.

However, it can be also seen from the table that the same fish pair D in experiment 4 does not produce peak positions in xTLMI and yTLMI with different signs. This latter observation indicates that the leading fish in xTLMI is the same leading fish in yTLMI. Our finding suggests that the role of information receiver/source for a fish pair is not necessarily constant in time. Similar observations of changes in roles can also be seen for fish pairs B, F, and L. From our experimental observations, fish pairs that exhibit anticipatory dynamics also display follow behaviors. However, the reverse is not true. One interesting observation from Table 2 is that interaction strength is smaller for anticipatory pairs.

### 3.5. DIF Detection by Statistical Methods

**Figure 7:**
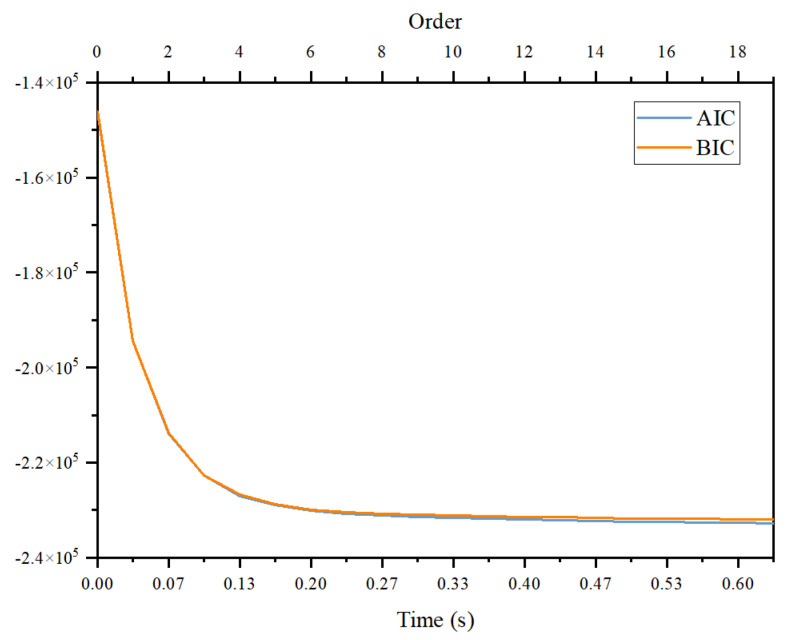
A typical estimation of order of auto-regressive model base on Bayesian information criterion (BIC) and Akaike information criterion (AIC) obtained from programs provided by the MVGC tool box. The order shown in the figure is related to the time step of our data of 1/30 s.

As mentioned above, three statistical methods are used to determine DIF by the time series obtained in the experiments. The results of GC test and Liang’s *T* test are summarized in Table 3. For the GC computation to obtain F , one needs to determine the order (lag) used in the computation of GC in the models of Equations (2) and (3). Figure 7 shows the order (time lag) dependence of the Akaike information criterion (AIC) and Bayesian information criterion (BIC) using the code of MVGC. Ideally, the order of the model is determined by the location of the minimum of the AIC/BIC curve. As can be seen from the figure, there seems to be no minimum, only saturation. The order shown in the table is determined automatically by MVGC. We have checked that if we fixed the order to be 10, some numerical values of the table will be changed, but the conclusion from Table 3 discussed below will not be changed.

**Figure 8:**
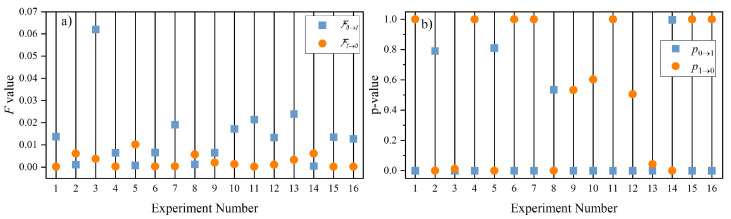
Results of F values from Granger causality based on the programs provided by MVGC. (**a**) F values for different experiments shown in Table 2 and (**b**) their responding *p*-value.

Figure 8a,b are the GC test results and the corresponding *p*-values from Table 3. A remarkable feature of Figure 8a is that all the $F_{0\to 1}>F_{1\to 0}$ for all the experiments except for experiment numbers 2, 5, 8, and 14. That means that the computed F values correctly predict that Fish 1 (follower) is receiving information from Fish 0 (leader) except for these four experiments. This difference is significant, as can also be seen from the *p*-value shown in Figure 8b.

The implication of the results of the GC test for experiments 2, 5, 8, and 14 is interesting because they are the cases for anticipatory dynamics. That means if we merely look at the x-component for their trajectories, one could have concluded that Fish 1 is the leader. It is only through the y-component of their trajectories that we conclude that these are cases for anticipatory dynamics; namely, information flow from Fish 0 to Fish 1, but Fish 1 is leading in action. Therefore, the use of the GC test to detect direction of information flow fails for anticipatory dynamics.

**Figure 9:**
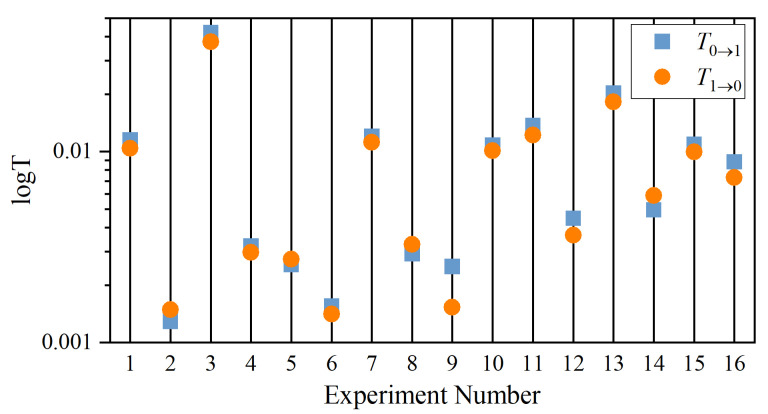
Results of Liang’s *T*-value computation from experiments shown in Table 2. It can be seen that $T_{0\to 1}$ are all larger than $T_{1\to 0}$ except for experiments 2, 5, 8, and 14, similar to the finding of using GC.

Following Liang [13], we have also computed $|T_{0\to 1}|$ and $|T_{1\to 0}|$ for our experimental dataset, and they are also shown in Table 2 and Figure 9. It is clear that all *T* are non-zero and all $|T_{0\to 1}|$ are close to $|T_{1\to 0}|$ . Therefore, there is no clear indication of causality. However, upon close examination, similar to the GC test, all the $|T_{0\to 1}|$ are larger than $|T_{1\to 0}|$ except for experiments 2, 5, 8, and 14. Although there is no clear indication of direction of information flow from using Liang’s analysis, our results show that Liang’s *T* method, similar to the GC test, fails to detect DIF in the cases of anticipatory dynamics.

We have also computed the TE between the two time series from the experiments. For the computation of TE from our experimental data, we need to first specify a history length that is similar to the order of the model in the method of GC. In the package of MVGC, the order is determined automatically by the package. However, for TE, we need to specify it manually when we use the python package PyInform to compute the TE. Since the number of permissible states in the system increases exponentially with history length, there will be a strong bias [16] because the data length is short or history length is long. For example, in our case of the trajectories in the X-direction divided into fiur states, the number of permissible states of the system will increase as $4^{h+1}$ , where *h* is the history length. For h=1,2,4 , and 8, the corresponding number of permissible states are 16,64,1024 , and 262,144, respectively. Since our data length is only 18,000, the results of TE computation will be unreliable when *h* is larger than 4. But in view of Figure 7, our history length should be chosen to be at least 5, which corresponds to a lag of about 165 ms.

**Figure 10:**
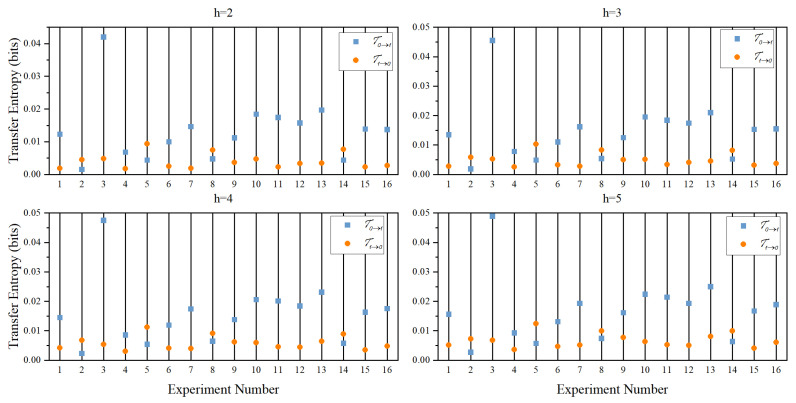
Computation of transfer entropy of experiments listed in Table 2 using the package PyInform. Here, *h* is the history length, and the two directions of transfer entropy are shown as blue and red. It can be seen that for most of the cases, TE is higher from 0 to 1, but the difference between the two directions are small. Similar to the computation of TLMI, the discretization here is also 2 bit, but a 4 bit discretization will give similar results but with different numerical values for the computed TE.

Figure 10 shows the results of TE computation of data from Table 3 for different history lengths from 2 to 5. It can be seen from the figure that, in general, $T_{0\to 1}$ is greater than $T_{1\to 0}$ , although their differences are small. This last finding shows that the DIF for most of the cases in the experiment are from 0 to 1. However, if we look closely at experiments 2, 5, 8, and 14, we find that $T_{0\to 1}$ is either very close to or smaller than $T_{1\to 0}$ . The last finding shows that the DIF for these cases are from 1 to 0. Therefore, the DIF obtained from the method of TE are the same as those determined by the GC test and Liang’s *T*. All these methods failed to detect the correct DIF for anticipatory dynamics.

### 3.6. Testing of DIF Detection with Simulated Anticipatory Data

The results from the three methods (GC, Liang’s *T*, and TE) are similar in that they all determine that the leading time series is detected as the information source and therefore fail to detect the possible anticipatory dynamics in experiments 2, 5, 8, and 14. To further demonstrate this property of these tools, we have also used simulated anticipatory dynamics for these tests. Our source signal, S(t) , is a lowpass Ornstein–Uhlenbeck time series that has been demonstrated to be able to produce anticipatory response from a negative group delay model [5]. The model of Equation (7) is then used to create the anticipatory responses Z(t) .

**Figure 11:**
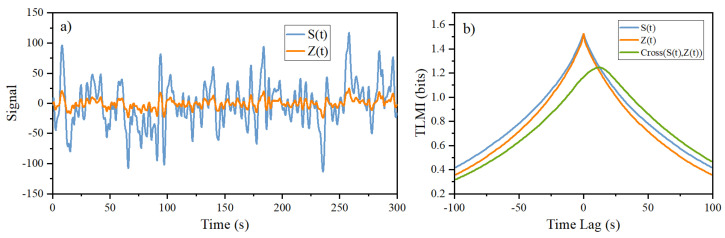
Simulation of anticipatory dynamics from Equation (7) with the following parameters: $\alpha =20s^{-1},k=5s^{-1}$ , and $t_{d}=1s$ . (**a**) The time course of lowpass OU signal, S(t) , and the output Z(t) . (**b**) The corresponding TLMI between S(t) and Z(t) . Here, S(t) is the lowpass ( 0.3 Hz) of W(t) with $\dot{W}\left(t\right)=-W\left(t\right)/\tau +\sigma \zeta \left(t\right)$ , where $\sigma =63.2s^{-1}$ , τ=1 , and ζ(t) is a dimensionless Gaussian white noise with zero mean and unit variance. Note that S(t) , Z(t) , and W(t) are all dimensionless, and both Equation (7) and W(t) are obtained by using simple Euler’s method with a time step size of 0.01 s. Similar results can also be obtained when a time step of 0.001 s is used. These results are obtained with a duration of 300 s of simulated time.

**Figure 12:**
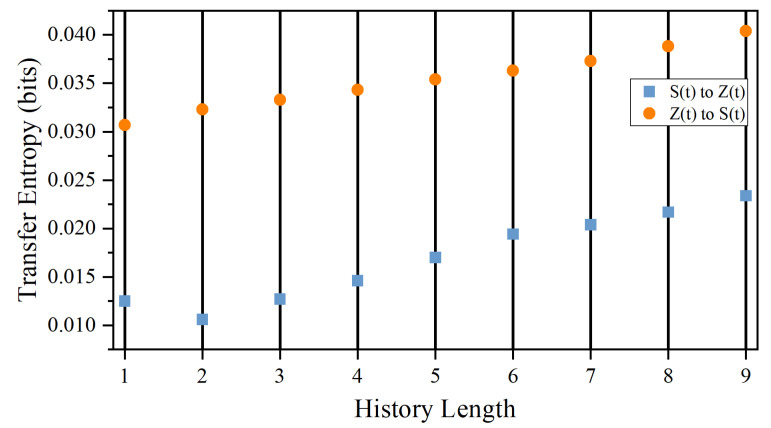
Measured TE between *S* and *Z* of the NGD model for various history length. It can be seen that TE from *Z* to *S* is always larger than that from the reverse direction. A history length is measured in a simulation step to be 0.01 s.

The simulation of Equation (7) and the corresponding TLMIs between S(t) and Z(t) are shown in Figure 11. From the peak position of the cross-TLMI, it is clear that Z(t) is leading S(t) . This is exactly the situation for anticipatory dynamics: information flow from S(t) to Z(t) while Z(t) is leading S(t) . When we perform the GC and the Liang’s *T* analysis with these two signals, we find that $F_{z\to s}>F_{s\to z}$ and $|T_{z\to s}\left|>\right|T_{s\to z}|$ . In other words, both GC and Liang’s method fail to detect the correct direction of information flow. Therefore, the failure of the detection of AD in experiments 2, 5, 8, and 14 does not mean that there is absence of AD in these experiments. We have also computed the corresponding TE of this NGD model for various history lengths, and the result is shown in Figure 12. It can be seen clearly from the figure that the direction of information flow is wrong because TE from *S* to *Z* is always larger than those from *Z* to *S*. Note that the discretization of the trajectories used in Figure 11b and Figure 12 are both 4 bit. Similar results can also be obtained when the discretization is changed to 2 bit.

## 4. Limitations

### 4.1. Predictive and True Causality

The argument we used above to obtain the direction of information flow (causality) between two time series is based on the improvement of prediction of future values of one time series by the inclusion of values of the other time series. The causality established in this manner is also known, more precisely, as predictive causality [17]. It is natural that we choose this methodology here because AD is predictive. In fact, all three methods used here to detect direction of information flow are predictive in nature. However, we would like to stress that predictive causality can be different from true causality [17], which requires a direct cause-and-effect mechanism [18] between two agents. Therefore, our results of detection of AD between a pair of fish should be taken with caution. We did not present any experimental evidence of a direct cause-and-effect mechanism between the two fish. Data needed to establish true causality will require adequately designed intervention experiments [19]. The readers should keep in mind that anticipatory dynamics is predictive in nature and its detection does not imply that a biological cause-and-effect mechanism between the two fish has been inferred.

### 4.2. Detection of Anticipatory Dynamics

In this work, we are using the usual definition of AD in which information flows from the source to the receiver while the temporal order of the receiver is ahead of the source. In the case of a retina [4,5], it is clear that information flows from the visual stimulation to the retina. AD is said to be observed when the output the of the retina (receiver) is ahead of the stimulation (source). With this definition of AD, our claim of detection of AD is based on our observation that the motions of receiver fish is ahead of the source fish in some fish pairs. In our experiment, the source and receiver fish assignment is based on the passing order of the fish through the door and the assumption that the roles of the fish do not change during the experiment. However, the readers are reminded that these assumptions of the passing order and the constancy of the roles of the fish need to be further tested.

### 4.3. Issues of Entropy Based Causality

It is well known that one must be cautious when dealing with entropy, especially when it is used to deal with causality [20]. The counter-intuitive anticipatory dynamics under consideration here might even make matters worse. For example, in a simulation study of using TE to determine the direction of causality in coupled logistic maps with anticipatory properties, Hahs et al. [21] found that the direction of causality in this relatively simple case is dependent on the data resolution being used. Correct direction of causality can be found only when high enough data resolution is used to compute the transfer entropy. In fact, we have also checked that, for our simulation model above, the results could be quite different from that shown in Figure 12 if a higher data resolution is used in the computation of TE.

The two lines in Figure 12 will cross over at a certain history length when the data resolution is 6 bit or higher, suggesting that the direction of information flow is reversed. However, we find that this crossover can be removed by using longer simulated time (larger sample size). It is not clear if this phenomenon originates from the bias of sample size mentioned above or is related to the effect mentioned in Ref. [21]. Nevertheless, these results from the NGD model simulation clearly demonstrated that TE based causality must be taken with care. Unlike numerical simulations in which data resolution can be increased relatively easily, our experimental data resolution is fixed by the setup. The use of computed TE based causality in our experiments might not be conclusive. Of course, our assumption that the “driver” fish will go through the door first in the experiment also needs to be further tested.

### 4.4. Correlation and Causation

One common mistake in the study of causality between two time series is the confusion between correlation and causality. In Ref. [3], where anticipatory synchronization is considered, there is a master and a slave with identical intrinsic dynamics (before coupling). These two systems will synchronize with the slave system ahead of the master system. It would be easy to conclude that the slave system is the cause of the master system because its time course is ahead of that from the master. However, in such a case, there is no causal relation between them because, when the two systems are synchronized, there is no information flow between them. In fact, the coupling term vanishes when the two systems are synchronized. In such a case, these two systems are perfectly correlated, but there is no casual relation between them.

However, in the case of Ref. [2], the NGD model (Equation (7)) in our manuscript, S(t) and Z(t) are not only correlated but there is also a causal relation between them. Note that the intrinsic dynamics of Z(t) is purely relaxation without the coupling term. It is the coupling delayed feedback, $k(S\left(t\right)-Z\left(t-t_{d}\right))$ , which uses S(t) as the drive for the system Z(t) . The system Z(t) can produce anticipatory output of S(t) because of the negative group delay properties of system Z(t) as well as the predictability of signal S(t) by using a lowpass filter on a random signal. Had S(t) been totally random, there will be no anticipatory output from Z(t) . For this NGD model, the causal direction is clearly from S(t) to Z(t) . This simple NGD model cannot be used to understand the interaction between the fish pair. It is used here simply to demonstrate that the common tools for causality used here fail to detect the correct DIF when anticipatory data of known DIF are fed into these tools. In our experiments, there is no common external stimulation to the fish pair. Presumably, their correlated but not synchronized motions are the results of information exchanges between them.

### 4.5. Problem of Bi-Directional Coupling

It should be stated again that our definition of anticipatory dynamics is quite narrow. For example, if there are bi-directional anticipatory interactions between the two fish with almost equal strength, the resultant cross-TLMI will peak very close to zero time lag. In such a case, there will be no obvious leader in the two resultant time series and our method will reach the wrong conclusion that there is no AD. Therefore, our method of detecting AD probably cannot be used to detect causality in a closed-loop control system, such as the coupling between heart rate and respiration rate [22] or auto-regulation of blood pressure [23]. In a closed-loop control system, different variables of the system are coupled directly or indirectly in such a way to maintain the system in some desirable state. Therefore, the causal effects of different variables in such a system are investigated because these causal relations might reveal the underlying control mechanism. Methods such as Granger indices [24] have been used to infer the types of interaction between different variables in the controlled system. However, such an approach is probably not suitable for our investigation of the interaction between two fish. It is not clear if their trajectories can be regarded as the result of some kind of control. We could have also extended our NGD simulation model to have a bi-directional coupling. But as mentioned above, our detection method would probably fail.

## 5. Discussion

Traditionally, causality refers to the relationship between cause and effect, where a cause precedes an effect in time. It implies that the cause happens first, leading to the subsequent occurrence of the effect. However, as shown above in our simple numerical simulation, in the context of anticipatory dynamics, causality is not ordered by time. This happens because the responding system can incorporate future information of the source into its present behavior. Here, the future information of the source can be learned/anticipated by the responding system as in the case of NDG model, or it may use internal models or predictions to guide its behavior based on expected future conditions as in the case of anticipatory synchronization [3].

Our challenge is: how do we determine the causality between two given correlated time series as in our experiments in the context of anticipatory dynamics? From the discussion above, it is clear that the method of TLMI can be used to detect the temporal order of events between the two time series. Ideally, if DIF can also be computed from the two time series, one will be able to distinguish between the cases of “anticipatory” or “following”. However, it is non-trivial to determine DIF simply based on the data from the two times series as demonstrated by our experiment and simple simulation. In our experiments, we are able to tell the DIF because of the special design of using a door to determine which fish is the information source. Unfortunately, the methods of the GC test, Liang’s *T* analysis, and TE all failed to detect DIF for both experiments and our NGD generated data. These tools seem to detect DIF based on temporal order of events in the two time series. It is not clear if the existence of AD can be detected based only on the data from two time series. If yes, new tools need to be developed to detect it.

One interesting aspect of anticipatory dynamics is that the leading in time course does not necessary means the “leader” [25] is in control. In the phenomenon of anticipating synchronization demonstrated in Ref [3], it can be clearly seen that the “leader” (time course) can be the slave because the slave is actively anticipating the future movements of the master. It is known that when birds flock, the birds from low pecking order can lead (in the sense of time course) for some time. Perhaps, these low pecking order birds are just anticipating the future position/movement of their masters (birds of high pecking order). We are still in the early stages of understanding these emerging behaviors from these active entities in the sense they are not only self-propelling but at the same time making active decisions. Our experimental finding that most of the fish pairs are interacting by “following” dynamics suggests that during the schooling of fish, perhaps, most of the fish are just following the trajectories of their neighbors. It would then be interesting to investigate if the direction of movement of a school of fish is determined by some leaders or it is just spontaneously reacting to the environment such as the currents in the sea or the location of prey and/or predators.

Finally, we would like to note that the method of analysis presented here can be further tested by using zebrafish with known altered behavior; for example, fish with neuro-degenerative disease [26]. Presumably, the dynamics of interaction between these fish might be quite different because of their impaired information processing capability.

## Figures and Tables

**Table 1:** Probability of the choice of doors by the fish measured in four experiments.

Experiment Number	Number of Passages		
	**Left**	**Middle**	**Right**
1	28	19	33
2	28	20	20
3	51	16	31
4	20	6	18
Probability	43.8%	21.0%	35.2%

**Table 2:** Summary of cross-TLMI of experimental data. There are a total of 16 experiments (Expt) reported, but there are only 12 pairs of fish. The peak height is reported as the ratio between peak heights from the cross and auto-TLMI. The decay time is taken as the half decay time of the auto-TLMI.

		Properties of Cross xTLMI			Properties of Cross yTLMI		
**Expt Number**	**Fish Pair**	**Peak** **Position(s)**	**Peak Height**	**Decay Time(s)**	**Peak** **Position(s)**	**Peak Height**	**Decay Time(s)**
1	A	−0.83	0.22	0.57	−1.10	0.41	6.13
2	B	0.63	0.09	0.53	−3.87	0.35	7.90
3	B	−0.23	0.31	0.53	−0.53	0.48	6.03
4	C	−0.60	0.12	0.87	−0.73	0.19	3.97
5	D	0.50	0.09	0.47	−3.30	0.28	9.30
6	D	−0.63	0.12	0.60	−11.17	0.20	19.97
7	E	−0.77	0.20	0.40	−1.43	0.36	19.97
8	F	0.47	0.13	0.50	−0.53	0.26	2.53
9	F	−0.27	0.16	0.53	−1.13	0.19	2.47
10	G	−0.63	0.22	0.63	−1.73	0.39	13.47
11	H	−0.60	0.26	0.63	−1.37	0.26	4.67
12	I	−0.30	0.17	1.23	−0.67	0.22	7.40
13	J	−0.43	0.25	0.47	−0.53	0.34	4.93
14	L	0.57	0.23	0.83	−0.33	0.30	17.53
15	L	−0.67	0.27	1.03	−1.33	0.31	9.47
16	M	−0.40	0.21	0.87	−1.43	0.23	3.73

**Table 3:** Summary of GC test and Liang’s *T* using data from experiments listed in Table 2.

Expt Number	Fish	Order	*F* (MVGC)		*p*-Value (MVGC)		Liang’s *T*	
	**Pair**	**(BIC)**	$F_{0\to 1}$	$F_{1\to 0}$	$p_{0\to 1}$	$p_{1\to 0}$	$T_{0\to 1}$	$T_{1\to 0}$
1	A	20	$1.37\times 10^{-2}$	$1.90\times 10^{-4}$	$0.00\times 10^{0}$	$1.00\times 10^{0}$	$1.15\times 10^{-2}$	$1.04\times 10^{-2}$
2	B	20	$1.17\times 10^{-3}$	$6.13\times 10^{-3}$	$7.90\times 10^{-3}$	$4.61\times 10^{-7}$	$1.29\times 10^{-3}$	$1.49\times 10^{-3}$
3	B	20	$6.20\times 10^{-2}$	$3.78\times 10^{-3}$	$0.00\times 10^{0}$	$1.25\times 10^{-2}$	$4.20\times 10^{-2}$	$3.75\times 10^{-2}$
4	C	15	$6.48\times 10^{-3}$	$2.64\times 10^{-4}$	$1.43\times 10^{-7}$	$1.00\times 10^{0}$	$3.20\times 10^{-3}$	$2.97\times 10^{-3}$
5	D	11	$7.89\times 10^{-4}$	$1.02\times 10^{-2}$	$8.10\times 10^{-1}$	$2.22\times 10^{-16}$	$2.56\times 10^{-3}$	$2.73\times 10^{-3}$
6	D	18	$6.55\times 10^{-3}$	$3.15\times 10^{-4}$	$2.14\times 10^{-8}$	$1.00\times 10^{0}$	$1.55\times 10^{-3}$	$1.41\times 10^{-3}$
7	E	20	$19.91\times 10^{-2}$	$4.01\times 10^{-4}$	$0.00\times 10^{0}$	$1.00\times 10^{0}$	$1.20\times 10^{-2}$	$1.12\times 10^{-2}$
8	F	14	$1.26\times 10^{-3}$	$5.66\times 10^{-3}$	$5.35\times 10^{-1}$	$2.03\times 10^{-7}$	$2.92\times 10^{-3}$	$3.27\times 10^{-3}$
9	F	20	$6.45\times 10^{-3}$	$2.03\times 10^{-3}$	$6.01\times 10^{-6}$	$5.32\times 10^{-1}$	$2.50\times 10^{-3}$	$1.53\times 10^{-3}$
10	G	20	$1.72\times 10^{-2}$	$1.39\times 10^{-3}$	$0.00\times 10^{0}$	$6.03\times 10^{-1}$	$1.08\times 10^{-2}$	$1.01\times 10^{-2}$
11	H	20	$2.14\times 10^{-2}$	$1.77\times 10^{-4}$	$0.00\times 10^{0}$	$1.00\times 10^{0}$	$1.37\times 10^{-2}$	$1.22\times 10^{-2}$
12	I	10	$1.33\times 10^{-2}$	$1.07\times 10^{-3}$	$0.00\times 10^{0}$	$5.05\times 10^{-1}$	$4.48\times 10^{-3}$	$3.65\times 10^{-3}$
13	J	20	$2.39\times 10^{-2}$	$3.27\times 10^{-2}$	$0.00\times 10^{0}$	$4.35\times 10^{-2}$	$2.03\times 10^{-2}$	$1.82\times 10^{-2}$
14	L	10	$5.07\times 10^{-4}$	$6.16\times 10^{-3}$	$9.96\times 10^{-1}$	$3.30\times 10^{-8}$	$4.95\times 10^{-3}$	$5.89\times 10^{-3}$
15	L	10	$1.35\times 10^{-2}$	$2.22\times 10^{-4}$	$0.00\times 10^{0}$	$1.00\times 10^{0}$	$1.09\times 10^{-2}$	$9.95\times 10^{-3}$
16	M	20	$1.27\times 10^{-2}$	$1.97\times 10^{-4}$	$1.11\times 10^{-16}$	$1.00\times 10^{0}$	$8.84\times 10^{-3}$	$7.32\times 10^{-3}$

## Data Availability

The datasets used or analyzed during the current study are available from the corresponding author on reasonable request.

## Abbreviations and Symbols

AD	Anticipatory dynamics
DIF	Direction of information flow
Expt	Experiment
GC	Granger causality
NGD	Negative group delay
OU	Ornstein–Uhlenbeck
TE	Transfer Entropy
xTLMI	Time lag mutual information from the x component
U,V	Time series from the trajectories
S,Z	Source and target time series in the NGD model
*W*	Ornstein–Uhlenbeck noise
*P*	Probability distribution
η,ϵ	Error (residuals)
F	F-test
*p*	*p*-value
*T*	Value of Liang’s *T* mnethod
*C*	Covariance
T	Value of transfer entropy
*H*	Entropy
